# Long-term effects of 12-month integrated weight-loss programme for children with excess body weight- who benefits most?

**DOI:** 10.3389/fendo.2023.1221343

**Published:** 2023-11-03

**Authors:** Joanna Szczyrska, Michał Brzeziński, Agnieszka Szlagatys-Sidorkiewicz

**Affiliations:** Department of Pediatrics, Gastroenterology, Allergology and Pediatric Nutrition, Medical University of Gdansk, Gdansk, Poland

**Keywords:** pediatric obesity, weight loss, long-term effects, lifestyle intervention weight loss programme, lifestyle, excess body weight, BMI reduction

## Abstract

The aim of the study was to assess long-term effects of the 12-month integrated weight-loss programme in children with excess body weight. We also attempted to identify the determinants of intervention effectiveness. Two groups were included in the analysis: 241 children with excess body weight who participated in the full 12-month intervention (full participation group) and 891 children with excess body weight who did not participate in the intervention (no participation group). Changes in BMI SDS, SBP SDS, DBP SDS and post-exercise HR with a follow-up period of 4 years between this groups were compared. In the full participation group, the reduction in mean BMI SDS was greater, we also observed significantly higher decrease in DBP SDS. Subgroup analysis by age category and sex showed a significant difference in the change in mean BMI SDS (from baseline to follow-up) in the subgroup of younger children and in the subgroup of younger girls. In the subgroup of younger girls significantly higher decrease in DBP SDS and SBP was also observed. Younger children, who participated in the intervention at age 6, particularly girls, benefited the most.

## 1 Introduction

In recent decades, the prevalence of excess body weight in children has increased rapidly. Although there has been a recent stabilization of excess body weight among children in several highly developed nations, the proportion of children affected by this health concern remains alarmingly high, and is on the rise in developing countries (1). According to the Non-communicable Disease Risk Factor Collaboration, the global prevalence of obesity in girls aged 5-19 increased from 0.7% in 1975 to 5.6% in 2016, and in boys from 0.9% to 7.8%. According to WHO data, in 2016, 41 million children under the age of 5 and 340 million children between the ages of 5 and 19 were overweight and obese (2). The WHO’s European Childhood Obesity Surveillance Initiative (COSI) study, conducted across 36 European countries between 2015 and 2017, revealed that nearly one in three children aged 7 to 9 years (28.7% of boys and 26.5% of girls) had overweight or obesity, and approximately one in ten of those children (12.5% of boys and 9% of girls) were classified as obese (3).

Numerous studies have demonstrated that children with excess body weight are at a greater risk of developing many diseases, particularly cardiovascular, metabolic, and mental disorders in comparison to those with normal body weight (4–8).

The majority of overweight and obese children become adults with obesity and its associated complications (9, 10), and some of these health issues may arise before they reach the age of 18 (5, 11, 12). This is why the WHO has recognized childhood obesity as one of the most significant public health challenges of the 21st century (13). It is now imperative to implement effective preventive and therapeutic measures for obesity in children. Decreasing body weight through effective interventions not only mitigates health consequences but also economic ones.

Numerous studies, meta-analyses, and systematic reviews have evaluated the efficacy of interventions used in children with excess body weight. Currently, multispecialty interventions aimed at lifestyle modification, which involve not only the children but also their parents or carers, are considered the recommended gold standard of treatment (14, 15). An example of this type of intervention is the 6-10-14 for Health programme implemented in 2011 in Gdansk, one of the major cities in Poland. In a study conducted on a group of 1100 overweight children aged 6-14 years, the short-term clinical effectiveness and cost-effectiveness of a 12-month multidisciplinary intervention implemented under this programme were demonstrated (16).

The aim of our study was to assess long-term effects of the 12-month integrated weight-loss programme in children with excess body weight, taking BMI, blood pressure and physical performance (cardiorespiratory fitness) into account. We also attempted to identify the determinants of intervention effectiveness.

## 2 Materials and methods

### 2.1 Study design

We performed the analysis of data collected from the ‘6-10-14 for Health’ health programme, which has been in operation in Gdansk since 2011. The primary objective of this programme is to introduce a 12-month integrated weight loss programme for children with overweight and obesity. The programme has two stages:

Stage I - screening tests conducted among primary and middle school students in Gdansk. The screening involves 3 age groups: 6 years old, 9-11 years old and 14 years old. The screening has been ongoing since 2011 up until now and data from the beginning up to 2018 is used in this study.

Stage II involves qualifying children with excess body weight (BMI ≥ 85th percentile) for the second stage of the programme, which comprises 12 months of comprehensive educational and health intervention. The intervention consists of four individual interdisciplinary consultations with a doctor, dietitian, physical activity specialist, and psychologist as per a 0-3-6-12 months schedule. The consultations aim to reduce body weight, change eating habits, modify physical activity patterns, and foster pro-health attitudes (17–19).

### 2.2 Participants

**Figure 1:**
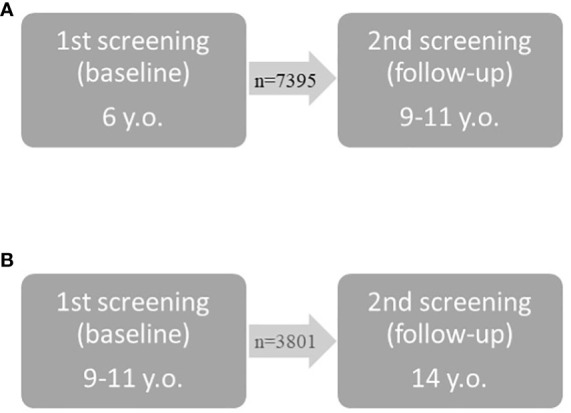
Screening points for younger **(A)** and older **(B)** children.

**Figure 2:**
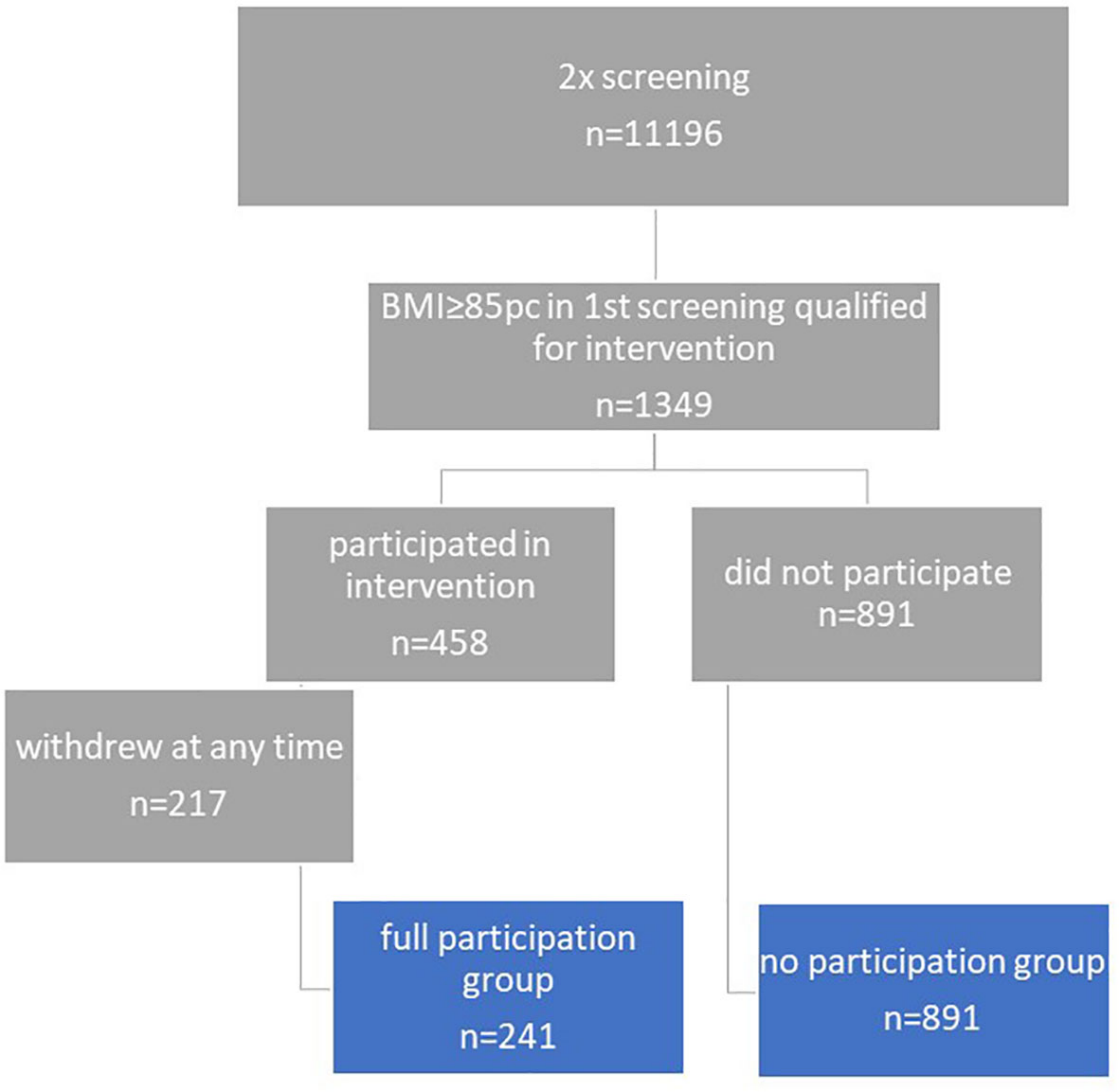
Study flow.

A group of 11 196 children was selected from the screening database of the ‘6-10-14 for Health’ programme, covering the years 2011 to 2018. These children underwent two assessments with a time interval of approximately 4 years between them, according to the programme’s general screening schedule. Younger children were assessed at 6 years old and then at 9-11 years old, while older children were assessed at 9-11 and 14 years old (as shown in **Figure 1**). During the first screening (baseline), excess body weight (BMI ≥85 percentile) was identified in 1349 (12.05%) children, which qualified them for stage II of the 12-month multidisciplinary intervention. Of this group of children, 241 completed the full 12-month programme consisting of four meetings, while 217 children started but dropped out at different stages, making this group too diverse for further analysis. On the other hand, 891 children who qualified for the intervention did not participate for various reasons unknown to the researchers. The study flow is presented in **Figure 2**.

Therefore, 2 groups were included in the analysis:

- Group I (full participation): inclusion criterion: baseline BMI≥ 85 percentile + participation in the full intervention, n= 241
- Group II (no participation): inclusion criterion: baseline BMI≥ 85 percentile + no intervention, n=891

### 2.3 Measurements

In all children, anthropometric and blood pressure measurements were taken and physical fitness was assessed at the first (baseline) and second (follow-up) assessment. In the group of younger children, the first assessment (baseline) was completed at 6 years old, and the second (follow-up) at the age of 9 to 11 years old. In the group of older children, the baseline assessment was completed at 9 to 11 years old, and the follow-up at 14 years old (**Figure 1**).

#### 2.3.1 Anthropometric measurements

Body height was measured in the Frankfurt position using a height gauge to the nearest 1 mm, and body weight was measured using a scale to the nearest 100 g. The measurements were taken with the children barefoot and wearing only underwear or gym clothes. The BMI (body mass index) was calculated based on the anthropometric measurements. In accordance with the criteria for diagnosing excess body weight in children in Poland, with reference to the OLAF national centile grids, a BMI≥85 and <=95th percentile was diagnosed as overweight and BMI≥95th percentile, as obesity (20).

#### 2.3.2 Blood pressure measurements

Blood pressure was measured using a sphygmomanometer (Omron) on the left arm with a properly sized cuff. The measurement was taken three times with the child in a sitting position, legs uncrossed, after a 5-minute rest. The average of the three measurements was recorded.

#### 2.3.3 Physical fitness assessment

To evaluate physical fitness, we utilized the Kasch Pulse Recovery Test (KPRT), a 3-minute step test. The test required participants to step up and down on a 0.305-metre-high step at a set cadence of 24 steps per minute for three minutes, followed by a one-minute and five-second rest in a sitting position. Throughout the test and recovery period, we continuously monitored the heart rate (HR) using the “Polar” electronic analyzer (Finland). The average post-exercise heart rate was recorded within one minute, starting five seconds after the test ended (21).

### 2.4 Ethics Approval

The study protocol was approved by Independent Bioethics Committee for Scientific Research of Medical University of Gdansk (decision no. NKBBN/228/2012 and NKBBN/228-197/2014). The study was registered in clinicaltrials.gov as an element of Community Based Obesity Prevention and Treatment Programme “6-10-14 for Health” NCT04143074.

### 2.5 Statistical analysis

A baseline characteristics of participants were reported as means, standard deviations and quartiles for quantitative data, whereas categorical variables were presented as counts and percentages. The between-group baseline characteristics differences were evaluated using the unpaired t-test for continuous data and the chi-square test for categorical variables.

Comparison of the percentage of children with a reduction in BMI SDS between study groups was analyzed using the chi-square test. Change from baseline was analyzed using the analysis of covariance (ANCOVA), where baseline values and observation time were treated as covariates. The least-square mean (LSM) and 95% confidence interval were calculated. The results of ANCOVA analysis were also shown in pre-specified subgroups: male, female, younger, and older. Linear regression was used to identify factors associated with change in BMI SDS from baseline to follow up. Variables were selected based on the forward selection procedure. Results were presented as beta coefficients, 95% confidence intervals, p-values, and adjusted coefficient of determination. No formal adjustment for multiple testing was made. The two-tailed tests were carried out at a significant level of 0.05. Statistical analysis was performed using the R statistical package (version 3.6.3).

## 3 Results

### 3.1 Basic characteristics

Data from the group of 241 children with excess body weight who participated in the full 12-month multidisciplinary intervention (full participation group) and the group of 891 children with excess body weight who did not participate in the intervention (no participation group) were compared. **Table 1** displays the basic characteristics of both groups. Children in the full participation group were older (mean age 8.47 ± 2.02 vs 8.13 ± 2.09, 0.023), had higher BMI (21.67 ± 2.57 vs 20.55 ± 2.41), BMI percentile (93.34 ± 3.82 vs 90.98 ± 4.17, and BMI SDS (1.56 ± 0.33 vs 1.38 ± 0.35), p<0.001.

**Table 1:** Characteristics of participants at baseline.

	Full participation (N=241)	No participation (N=891)	p-value
**Sex**			0.999
Female	122 (50.6%)	451 (50.6%)	
Male	119 (49.4%)	440 (49.4%)	
**Age category**			< 0.001
Older	119 (49.4%)	329 (36.9%)	
Younger	122 (50.6%)	562 (63.1%)	
**Age [in years]**			0.023
Mean (SD)	8.47 (2.02)	8.13 (2.09)	
Median (Q1,Q3)	7.33 (6.58, 10.42)	6.92 (6.50, 10.33)	
**BMI**			< 0.001
Mean (SD)	21.67 (2.57)	20.55 (2.41)	
Median (Q1,Q3)	21.50 (19.80, 22.80)	20.20 (18.55, 22.00)	
**BMI OLAF SDS**			< 0.001
Mean (SD)	1.56 (0.33)	1.38 (0.35)	
Median (Q1,Q3)	1.52 (1.30, 1.79)	1.28 (1.12, 1.54)	
**BMI OLAF**			< 0.001
Mean (SD)	1.56 (0.33)	1.38 (0.35)	
Median (Q1,Q3)	1.52 (1.30, 1.79)	1.28 (1.12, 1.54)	
**BMI percentile**			< 0.001
Mean (SD)	93.34 (3.82)	90.98 (4.17)	
Median (Q1,Q3)	94.00 (90.00, 97.00)	91.00 (87.00, 94.00)	
**BMI percentile categories**			< 0.001
overweight	131 (54.4%)	691 (77.6%)	
obesity	110 (45.6%)	200 (22.4%)	
**SBP**			0.905
Mean (SD)	106.77 (10.14)	106.86 (10.59)	
Median (Q1,Q3)	107.00 (100.00, 114.00)	106.00 (100.00, 113.00)	
**DBP**			0.696
Mean (SD)	68.92 (7.00)	69.12 (6.86)	
Median (Q1,Q3)	69.00 (64.00, 74.00)	69.00 (64.00, 73.00)	
**SBP SDS**			0.167
Mean (SD)	0.34 (0.90)	0.44 (0.94)	
Median (Q1,Q3)	0.45 (-0.20, 0.96)	0.44 (-0.15, 1.10)	
**DBP SDS**			0.237
Mean (SD)	1.09 (0.92)	1.17 (0.89)	
Median (Q1,Q3)	1.04 (0.48, 1.73)	1.18 (0.55, 1.75)	
**Hypertension**			0.928
No	166 (68.9%)	611 (68.6%)	
Yes	75 (31.1%)	280 (31.4%)	
**KPRT assessment**			0.050
Mean (SD)	2.76 (1.19)	2.95 (1.21)	
Median (Q1,Q3)	3.00 (2.00, 4.00)	3.00 (2.00, 4.00)	
**KPRT assessment categories**			0.111
Good (4-6)	51 (25.9%)	228 (31.8%)	
Poor (1-3)	146 (74.1%)	489 (68.2%)	
**Observation time**			< 0.001
Mean (SD)	3.73 (0.93)	3.47 (0.96)	
Median (Q1,Q3)	3.83 (3.00, 4.50)	3.42 (2.79, 4.16)	

Based on the BMI percentile criteria, 54.4% of children in the full participation group were overweight (BMI 85-95 percentile) and 45.6% had obesity (BMI ≥ 95 percentile), while in the no participation group, overweight was diagnosed in 77.6% of the children, and obesity in 22.4% (p<0.001). The mean time from baseline to follow up was 3.53 ± 0.96 years and was longer in the full participation group (3.73 ± 0.93 vs 3.47± 0.96, p<0.001).

Due to the observed differences between full participation group and no participation group further analysis was performed by the use of covariance analysis (ANCOVA), where baseline values and observation time were treated as covariates, and additionally subgroup analysis based on age and sex.

### 3.2 Change in BMI SDS, SBP SDS, DBP SDS, and post-exercise heart rate during the observation period (from baseline to follow up)

A reduction in mean BMI SDS was observed in both study groups. However, in the full participation group, the reduction in mean BMI SDS was greater compared to the group of children not participating in the intervention -0.09 (-0.15,-0.02) vs -0.01 (-0.04, 0.02), p=0.04 (**Table 2**). A significantly higher decrease in DBP SDS was observed in the full participation group, compared to the no participation group: -0.45 (-0.58,-0.31) vs -0.22 (-0.29,-0.16) p=0.004 with the results adjusted for the initial DBP SDS value and observation time; -0.41 (-0.53,-0.28) vs -0.24 (-0.30,-0.17) p= 0.019 when also adjusted for age (**Table 3**).

**Table 2:** ANCOVA of change in BMI SDS from baseline to follow-up adjusted to baseline value, and observation time and adjusted to baseline value, age and observation time.

	Baseline		Follow-up		Change from baseline		
Group	N	Mean (SD)	N	Mean (SD)	N	Mean (SD)	LS Mean (95% CI)^a^
No participation	891	1.4 ( 0.35)	891	1.4 ( 0.56)	891	-0.0 ( 0.48)	-0.01 (-0.04, 0.02)
Full participation	241	1.6 ( 0.33)	241	1.4 ( 0.54)	241	-0.1 ( 0.46)	-0.09 (-0.15,-0.02)
Comparison			Difference in LS Mean (95% CI)^a^				p-value
Full participation - No participation			-0.07 (-0.14,-0.00)				0.040
^a^Based on an ANCOVA model after adjusting baseline BMI SDS and observation time. ANCOVA = Analysis of Covariance, CI = Confidence Interval, LS = Least Squares, SD = Standard Deviation							
	Baseline		Follow-up		Change from baseline		
Group	N	Mean (SD)	N	Mean (SD)	N	Mean (SD)	LS Mean (95% CI)^b^
No participation	891	1.4 ( 0.35)	891	1.4 ( 0.56)	891	-0.0 ( 0.48)	-0.01 (-0.04, 0.02)
Full participation	241	1.6 ( 0.33)	241	1.4 ( 0.54)	241	-0.1 ( 0.46)	-0.09 (-0.15,-0.02)
Comparison			Difference in LS Mean (95% CI)^b^				p-value
Full participation - No participation			-0.07 (-0.14,-0.00)				0.040
^b^Based on an ANCOVA model after adjusting baseline BMI SDS, age and observation time. ANCOVA = Analysis of Covariance, CI = Confidence Interval, LS = Least Squares, SD = Standard Deviation							

**Table 3:** ANCOVA of change in DBP SDS from baseline to follow-up adjusted to baseline value, and observation time and adjusted to baseline value, age and observation time.

	Baseline		Follow-up		Change from baseline		
Group	N	Mean (SD)	N	Mean (SD)	N	Mean (SD)	LS Mean (95% CI)^a^
No participation	891	1.2 ( 0.89)	891	0.9 ( 1.07)	891	-0.2 ( 1.22)	-0.22 (-0.29,-0.16)
Full participation	241	1.1 ( 0.92)	241	0.7 ( 1.09)	241	-0.4 ( 1.26)	-0.45 (-0.58,-0.31)
Comparison			Difference in LS Mean (95% CI)^a^				p-value
Full participation - No participation			-0.22 (-0.37,-0.07)				.004
^a^Based on an ANCOVA model after adjusting baseline DBP SDS and observation time. ANCOVA = Analysis of Covariance, CI = Confidence Interval, LS = Least Squares, SD = Standard Deviation							
	Baseline		Follow-up		Change from baseline		
Group	N	Mean (SD)	N	Mean (SD)	N	Mean (SD)	LS Mean (95% CI)^b^
No participation	891	1.2 ( 0.89)	891	0.9 ( 1.07)	891	-0.2 ( 1.22)	-0.24 (-0.30,-0.17)
Full participation	241	1.1 ( 0.92)	241	0.7 ( 1.09)	241	-0.4 ( 1.26)	-0.41 (-0.53,-0.28)
Comparison			Difference in LS Mean (95% CI)^b^				p-value
Full participation - No participation			-0.17 (-0.32,-0.03)				0.019
^b^Based on an ANCOVA model after adjusting baseline DBP SDS, age and observation time. ANCOVA = Analysis of Covariance, CI = Confidence Interval, LS = Least Squares, SD = Standard Deviation							

No significant difference in the change in mean SBP SDS and post-exercise heart rate was observed between the compared groups (**Tables 4**, **5**).

**Table 4:** ANCOVA of change in SBP SDS from baseline to follow-up adjusted to baseline value, and observation time and adjusted to baseline value, age and observation time.

	Baseline		Follow-up		Change from baseline		
Group	N	Mean (SD)	N	Mean (SD)	N	Mean (SD)	LS Mean (95% CI)^a^
No participation	891	0.4 ( 0.94)	891	0.6 ( 1.07)	891	0.1 ( 1.16)	0.15 ( 0.09, 0.22)
Full participation	241	0.3 ( 0.90)	241	0.6 ( 1.11)	241	0.2 ( 1.17)	0.15 ( 0.02, 0.28)
Comparison			Difference in LS Mean (95% CI)^a^				p-value
Full participation - No participation			0.00 (-0.14, 0.15)				0.992
^a^Based on an ANCOVA model after adjusting baseline SBP SDS and observation time. ANCOVA = Analysis of Covariance, CI = Confidence Interval, LS = Least Squares, SD = Standard Deviation							
	Baseline		Follow-up		Change from baseline		
Group	N	Mean (SD)	N	Mean (SD)	N	Mean (SD)	LS Mean (95% CI)^b^
No participation	891	0.4 ( 0.94)	891	0.6 ( 1.07)	891	0.1 ( 1.16)	0.17 ( 0.10, 0.23)
Full participation	241	0.3 ( 0.90)	241	0.6 ( 1.11)	241	0.2 ( 1.17)	0.10 (-0.02, 0.22)
Comparison			Difference in LS Mean (95% CI)^b^				p-value
Full participation - No participation			-0.06 (-0.20, 0.07)				0.368
^b^Based on an ANCOVA model after adjusting baseline SBP SDS, age and observation time. ANCOVA = Analysis of Covariance, CI = Confidence Interval, LS = Least Squares, SD = Standard Deviation							

**Table 5:** ANCOVA of change in post-exercise HR (KPRT) from baseline to follow-up adjusted to baseline value, and observation time and adjusted to baseline value, age and observation time.

	Baseline		Follow-up		Change from baseline		
Group	N	Mean (SD)	N	Mean (SD)	N	Mean (SD)	LS Mean (95% CI)^a^
No participation	536	121.6 (14.82)	536	119.4 (18.06)	536	-2.2 (19.29)	-2.60 (-4.11,-1.09)
Full participation	154	123.6 (20.23)	154	121.2 (18.88)	154	-2.4 (27.12)	-1.20 (-4.04, 1.63)
Comparison			Difference in LS Mean (95% CI)^a^				p-value
Full participation - No participation			1.39 (-1.83, 4.62)				0.398
^a^Based on an ANCOVA model after adjusting baseline KPRT and observation time. ANCOVA = Analysis of Covariance, CI = Confidence Interval, LS = Least Squares, SD = Standard Deviation							
	Baseline		Follow-up		Change from baseline		
Group	N	Mean (SD)	N	Mean (SD)	N	Mean (SD)	LS Mean (95% CI)^b^
No participation	536	121.6 (14.82)	536	119.4 (18.06)	536	-2.2 (19.29)	-2.63 (-4.14,-1.12)
Full participation	154	123.6 (20.23)	154	121.2 (18.88)	154	-2.4 (27.12)	-1.09 (-3.94, 1.75)
Comparison			Difference in LS Mean (95% CI)^b^				p-value
Full participation - No participation			1.53 (-1.70, 4.77)				0.353
^b^Based on an ANCOVA model after adjusting baseline KPRT, age and observation time. ANCOVA = Analysis of Covariance, CI = Confidence Interval, LS = Least Squares, SD = Standard Deviation							

**Figure 3:**
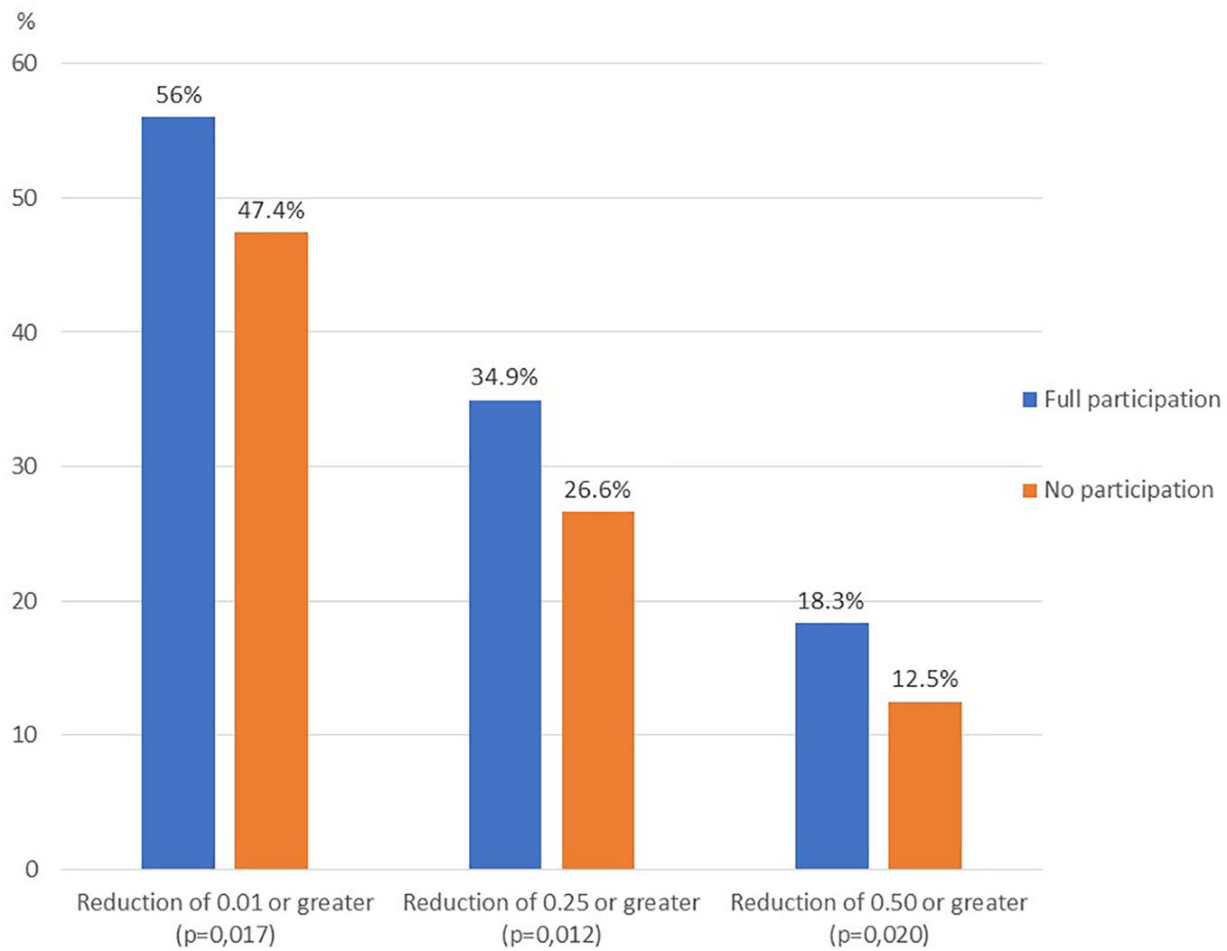
BMI SDS reduction of ≥ 0.01, ≥ 0.25 and ≥ 0.50 from baseline to follow up - full participation vs no participation comparison.

In the group of children participating in the full intervention, a reduction in BMI SDS was found in 56% of children, with 35% reducing their BMI SDS by at least 0.25, and 18% by 0.5 or more. In the group of children who did not participate in the intervention at all, the percentage of children with such a reduction in BMI SDS was significantly lower (**Figure 3**).

Subgroup analysis by age category and sex showed a significant difference in the change in BMI SDS (from baseline to follow-up) between children who participated in the full intervention and non-participating children in the subgroup of younger children and in the subgroup of younger girls. Furthermore, there was a change in mean DBP SDS in the subgroups of girls, boys, younger girls, and older boys. There was also a significant difference in the change in SBP SDS, but only in the subgroup of younger girls (**Table 6**).

**Table 6:** Statistically significant results of ANCOVA analysis of changes from baseline to follow-up in subgroups by age and sex.

Subpopulation: Younger							
ANCOVA of change in BMI SDS from baseline to follow-up							
Group	Baseline		Follow-up		Change from baseline		
	N	Mean (SD)	N	Mean (SD)	N	Mean (SD)	LS Mean (95% CI)^a^
No participation	562	1.4 (0.38)	562	1.4 (0.51)	562	0.0 (0.41)	0.01 (-0.03, 0.04)
Full participation	122	1.6 (0.35)	122	1.5 (0.49)	122	-0.1 (0.40)	-0.10 (-0.17,-0.03)
Comparison			Difference in LS Mean (95% CI)^a^				p-value
Full participation - No participation			-0.11 (-0.19,-0.03)				0.010
Subpopulation: Female							
ANCOVA of change in DBP SDS from baseline to follow-up							
Group	Baseline		Follow-up		Change from baseline		
	N	Mean (SD)	N	Mean (SD)	N	Mean (SD)	LS Mean (95% CI)^a^
No participation	451	1.1 (0.91)	451	0.9 (1.05)	451	-0.2 (1.23)	-0.20 (-0.29,-0.11)
Full participation	122	1.1 (0.93)	122	0.7 (0.96)	122	-0.4 (1.16)	-0.41 (-0.59,-0.24)
Comparison			Difference in LS Mean (95% CI)^a^				p-value
Full participation - No participation			-0.21 (-0.41,-0.01)				0.038
Subpopulation: Male							
ANCOVA of change in DBP SDS from baseline to follow-up							
Group	Baseline		Follow-up		Change from baseline		
	N	Mean (SD)	N	Mean (SD)	N	Mean (SD)	LS Mean (95% CI)^a^
No participation	440	1.2 (0.87)	440	0.9 (1.09)	440	-0.3 (1.20)	-0.25 (-0.35,-0.15)
Full participation	119	1.1 (0.92)	119	0.7 (1.22)	119	-0.4 (1.36)	-0.48 (-0.68,-0.28)
Comparison			Difference in LS Mean (95% CI)^a^				p-value
Full participation - No participation			-0.23 (-0.45,-0.01)				0.041
Subpopulation: Younger & Female							
ANCOVA of change in BMI SDS from baseline to follow-up							
Group	Baseline		Follow-up		Change from baseline		
	N	Mean (SD)	N	Mean (SD)	N	Mean (SD)	LS Mean (95% CI)^a^
No participation	297	1.3 (0.40)	297	1.4 (0.55)	297	0.1 (0.43)	0.05 (0.00, 0.10)
Full participation	72	1.6 (0.35)	72	1.5 (0.52)	72	-0.1 (0.40)	-0.08 (-0.18, 0.02)
Comparison			Difference in LS Mean (95% CI)^a^				p-value
Full participation - No participation			-0.13 (-0.24,-0.02)				0.025
ANCOVA of change in DBP SDS from baseline to follow-up							
Group	Baseline		Follow-up		Change from baseline		
	N	Mean (SD)	N	Mean (SD)	N	Mean (SD)	LS Mean (95% CI)^a^
No participation	297	1.1 (0.86)	297	1.1 (0.96)	297	-0.0 (1.18)	0.00 (-0.10, 0.11)
Full participation	72	1.1 (0.82)	72	0.9 (0.94)	72	-0.2 (1.09)	-0.25 (-0.46,-0.03)
Comparison			Difference in LS Mean (95% CI)^a^				p-value
Full participation - No participation			-0.25 (-0.49,-0.01)				0.043
ANCOVA of change in SBP SDS from baseline to follow-up							
Group	Baseline		Follow-up		Change from baseline		
	N	Mean (SD)	N	Mean (SD)	N	Mean (SD)	LS Mean (95% CI)^a^
No participation	297	0.3 (0.90)	297	0.3 (0.99)	297	0.0 (1.14)	0.02 (-0.09, 0.13)
Full participation	72	0.2 (0.86)	72	-0.0 (1.02)	72	-0.2 (1.13)	-0.26 (-0.48,-0.03)
Comparison			Difference in LS Mean (95% CI)^a^				p-value
Full participation - No participation			-0.27 (-0.52,-0.03)				0.031
Subpopulation: Older & Male							
ANCOVA of change in DBP SDS from baseline to follow-up							
Group	Baseline		Follow-up		Change from baseline		
	N	Mean (SD)	N	Mean (SD)	N	Mean (SD)	LS Mean (95% CI)^a^
No participation	175	1.1 (0.91)	175	0.6 (1.15)	175	-0.6 (1.24)	-0.56 (-0.72,-0.40)
Full participation	69	1.1 (0.91)	69	0.3 (1.17)	69	-0.8 (1.23)	-0.88 (-1.14,-0.62)
Comparison			Difference in LS Mean (95% CI)^a^				p-value
Full participation - No participation			-0.32 (-0.62,-0.01)				0.042

a Based on an ANCOVA model after adjusting baseline value and observation time.

ANCOVA, Analysis of Covariance; CI, Confidence Interval; LS, Least Squares; SD, Standard Deviation.

However, there were no significant differences in the change in mean post-exercise heart rate between children who participated in the full intervention and those who did not participate, in any of the analyzed subgroups.

**Table 6** presents only statistically significant results of the subgroup analysis, while the entire analysis in subgroups is included in **Supplementary Materials**.

**Figure 4:**
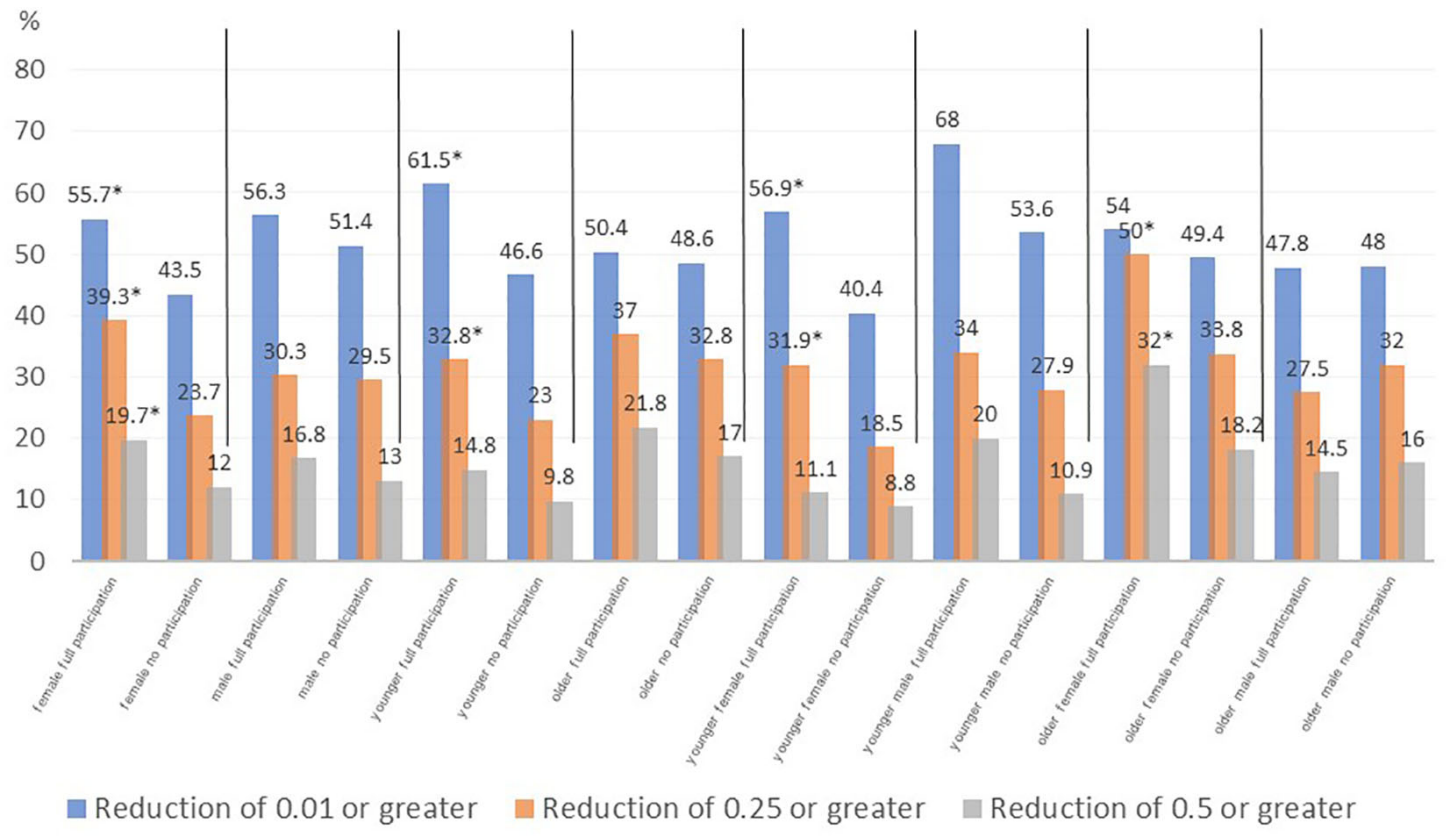
BMI SDS reduction from baseline to follow up of ≥ 0.01, ≥ 0.25 and ≥ 0.50 - full participation vs no participation comparison in subpopulations by age and sex (*- p<0.05 full participation vs no participation).

Comparisons of the percentage of children with a reduction in of BMI SDS ≥0.01, ≥0.25, ≥ 0.5, respectively, across subgroups, are shown in **Figure 4**.

A multivariate regression analysis conducted in the full intervention group did not find any significant relationship between the extent of change in BMI SDS (from baseline to follow up) and sex, age group, baseline BMI SDS, baseline physical fitness assessment, and baseline blood pressure. However, in the non-participating children group, there was a significant but weak relationship between the change in BMI SDS (from baseline to follow up) and the baseline BMI SDS (beta coefficient -0.162, p= 0.002) (**Table 7**).

**Table 7:** Multivariate linear regression of predictors of change in BMI SDS from baseline to follow-up.

Full participation					
	Beta coefficient	95% CI lower	95% CI upper	p-value	Adjusted R^2^
(Intercept)	-0.018	-0.458	0.421	0.936	0.048
BMI OLAF SDS at baseline	-0.063	-0.258	0.132	0.523	
Sex Male	0.058	-0.071	0.188	0.377	
Age category Younger	-0.053	-0.184	0.078	0.423	
Hypertension Yes	-0.128	-0.270	0.014	0.077	
KPRT assessment categories good	-0.128	-0.273	0.018	0.086	
Observation time	0.033	-0.040	0.105	0.376	
No participation					
	Beta coefficient	95% CI lower	95% CI upper	p-value	Adjusted R^2^
(Intercept)	0.337	0.137	0.538	0.001	0.023
BMI SDS baseline	-0.162	-0.265	-0.059	0.002	
Sex Male	-0.052	-0.121	0.016	0.134	
Age category Younger	-0.008	-0.081	0.065	0.832	
Hypertension Yes	-0.005	-0.081	0.072	0.900	
KPRT assessment categories good	-0.047	-0.123	0.029	0.223	
Observation time	-0.015	-0.052	0.021	0.416	

## 4 Discussion

Preventing and treating obesity and its complications in children is an important and difficult task. Various approaches can be employed to achieve these goals, including lifestyle modifications, medication, and bariatric surgery. Multidisciplinary behavioral interventions have shown promising results in promoting weight loss in children of all ages, as evidenced by meta-analyses (22–24). Lifestyle interventions are currently the recommended and the most commonly used method for treating childhood obesity, while the evidence for pharmacological and surgical treatment is still too limited to fully conclude on their efficacy and safety (14, 22, 25). However, it has still not been established which lifestyle intervention is most effective. Systematic reviews suggest a greater effectiveness of multidisciplinary interventions (ideally, involving professionals), involving the family (especially in children <12 years old) and with a longer duration (26–29). All these conditions are met by the intervention presented in this analysis and implemented as part of the ‘6-10-14 for Health’ programme.

Short-term studies are the most common in the literature that assesses the effectiveness of interventions in children with excess body weight, and evaluate outcomes immediately after intervention completion. Although longer-term studies exist, the follow-up period does not typically exceed two years. Due to variations in intervention types and durations, time between intervention and follow-up, and study designs, comparing results across studies can be challenging.

Obesity is known to be a chronic and recurrent disorder and despite the evidence for the short-term effectiveness of the interventions used, the sustainability of the changes achieved is important. In our study, we assessed long-term changes in BMI, blood pressure and physical fitness in a large group of children after a 4-year follow-up period. We compared children who participated in the course of the full 12-month intervention in ‘6-10-14 for Health’ programme to those who qualified for the intervention but did not participate.

When analyzing the effectiveness of lifestyle interventions in children with excess body weight, the question of when we can conclude that the intervention is effective might arise. From a medical point of view, the most important goal is to reduce cardiovascular risk factors and the risk of comorbidities. Based on the available studies, it is not possible to clearly determine the size of the clinically significant change in SDS BMI in children, i.e. a change sufficient to reduce cardiovascular risk. In adults, weight loss *≥* 5% is indicated in a number of guidelines for weight reduction as having a beneficial effect on a number of cardiovascular risk factors (30–32). Kolsgaard et al. reported that in children, even a slight reduction in BMI z-score (0.00-0.10) was associated with an improvement in insulin resistance homeostatic model assessment (HOMA- IR), reducing the risk of diabetes and cardiovascular disease (33). Several studies in children reported decreases in SDS BMI *≥* 0.25 as significant for reducing cardiovascular risk factors, with a reduction in SDS BMI *≥* 0.5 significantly increasing these health benefits (34–37). Weiss et al. estimated that a reduction in BMI SDS *≥* 0.09 was associated with an increase in HDL and a decrease in glucose and triglyceride levels. They also observed an increase in cardiovascular risk in children with an increase in obesity (38). Achieving improvements in quality of life is an important goal in the treatment of obesity, particularly from the patient’s perspective. Studies on children with obesity who participate in interventions have shown that quality of life can improve irrespective of weight reduction (39–41). However, the study authors emphasise that there is a need for further research in this area, necessary to determine the optimal duration and intensity of interventions (26). In our study, 56% of children participating in the full intervention reduced their BMI z-score, more than a third by at least 0.25, and 18% achieved a reduction in their BMI z-score of at least 0.5, significantly more than in the group of children not participating in the intervention. We also observed a favorable change in blood pressure in children participating in the full intervention. The reduction in DBP SDS was significantly higher in the full participation group than in children not participating in the intervention. In the subgroup of younger girls, we also observed a significant difference in the change in SBP SDS. Research in children on this issue is limited, but studies in adults show that even a small reduction in DBP (2 mmHg in the population distribution mean) is associated with a 17% reduction in the incidence of hypertension, as well as a 6% reduction in cardiovascular risk and a 15% reduction in the risk of stroke and TIA (42). Reducing blood pressure in children with excess body weight appears thus to offer a significant long-term health benefit, especially in the context of cardiovascular risk.

A Cochrane meta-analysis evaluating the effectiveness of interventions in children aged 6-11 years old with overweight and obesity (based on an analysis of 70 randomized studies) found that multi-component behavioral interventions that include diet, physical activity and behavioral changes can be beneficial for achieving reductions in BMI, BMI z-score and body weight in children, with the mean difference in change in BMI z-score between the intervention and control groups amounting to -0.06 (95% CI -0.10, -0.02). The findings of this meta-analysis suggest that interventions are effective at the time of completion and up to 6 months after the intervention. However, the authors acknowledged that sustaining the effects of interventions in the long term may be challenging, not necessarily due to the intervention’s failure, but rather due to a lack of maintenance interventions (23). Our study also found a significant reduction in BMI SDS, similar to what was reported in the meta-analysis. It is worth emphasizing that the reduction in BMI SDS observed in our study was sustained in the long term, with a follow-up period of 4 years.

In the group of children participating in the full intervention, multivariate regression analysis did not show a significant relationship between the change (reduction in BMI SDS) and other factors that would have predicted the efficacy of the intervention before it started. However, subgroup analysis by age and sex revealed a statistically significant difference between children participating in the full intervention and not participating in some of these subgroups only. The reduction in mean BMI SDS was significantly greater in the subgroup of younger children participating in the full intervention: - 0.10 (95% CI -0.17, -0.03) compared to non-participating children, whose mean BMI SDS remained essentially unchanged over the follow-up period at +0.01 (95% CI -0.03, 0.04). In the subgroup of older children, however, in both the full intervention and non-participation groups, we observed a reduction in mean BMI SDS: full participation -0.08 (95% CI -0.18, 0.03) vs no participation -0.04 (95% CI -0.11, 0.02), the difference between intervention and no-intervention groups was not statistically significant. These results are in line with the available literature. Most studies indicate greater long-term effectiveness of interventions in younger children. Wiegand et al. demonstrated that children aged 5-11 years were more likely to achieve weight reduction than 12- to 15-year-olds (43), and Reinehr et al. indicated greater effectiveness of interventions in children younger than 8 years (44).

It is worth noting that in the subgroup of younger girls, significant differences between the children participating in the full intervention and those not participating concerned not only the changes in mean BMI SDS, but also in blood pressure, both DBP SDS and SBP SDS, suggesting that 6-year-old girls participating in the intervention appear to benefit the most from the applied intervention.

As mentioned above, the most important goal, especially long-term, of treating childhood obesity is to reduce cardiovascular risk and prevent the development of comorbidities. This risk increases with the duration of obesity (45). The concept of “obesity years” has been introduced in the literature as a measure of the degree and duration of obesity. This factor is strongly associated with risk of developing type 2 diabetes, but also an increased cardiovascular risk (46). The more obesity years, the higher the risk of complications, which is why it is so important to reduce this factor. Effective interventions at a younger age can reduce the number of years with obesity and thus reduces the risk of developing complications associated with obesity.

Despite the strengths of the study, including a large number of participating children from a single centre, and a long follow-up period, we acknowledge its limitations. The study was not a randomised trial and the group of non-participating children was only a comparison group and not a typical control group. Study groups were selected from the screening database of the “6-10-14 for Health” programme. The programme criteria were open for every town citizen in a fitting age group (screening tests were conducted in primary and middle schools) and everyone willing could take part. Moreover, interventional part had only one inclusion criteria: excessive body weight. Of course, if someone was not willing to take part, we had no means to change their decision- the programme was open, but not mandatory. This could have possibly creating a sample selection bias. This model created also a huge non- participation and considerable dropout groups- guardians of 891 of the selected patients did not answer our invitation and 217 resigned at some point. Reasons for both started but dropped out at different stages or not taking part at all could be numerous, some of which may have created a bias, especially in non- participating group. The patients had no obligation to declare reason for not taking part or leaving and were not asked to as this program was to be as friendly and as easy on the patient as possible, which makes the assessment of the bias hard to perform. There may have existed some unmeasured and unassessed confounding factors, such as the level of motivation, awareness of the health problem or parental education, or the use of other forms of treatment and support. One has also to remember that studied group consisted of patients that took part in the program twice at different age points. Due to the long follow-up which helps us to assess long-term effects of intervention a question of time influence on patients’ behavior also arises- some patients or their families could have changed their views on obesity and the three-year-long enrollment span could also be long enough for the society to change their views on obesity, although we find this unlikely.

It is worth emphasising that the data analysed in the study were collected in the course of day-to-day clinical practice and reflect the real-life circumstances of managing overweight and obesity in children. And also therefore results of our study give real- world evidence for long term effects of intervention for children with excess body weight.

## 5 Conclusion

Participation in the full 12-month intervention within the ‘6-10-14 for Health’ programme resulted in a greater long-term reduction in BMI SDS and blood pressure when compared to non-participating children. Younger children, who participated in the intervention at age 6, particularly girls, benefited the most.

The observed efficacy of the intervention in the younger age group and the relationship between the risk of developing complications and the duration of obesity suggest that intervention programmes should be targeted primarily at younger age group.

## Data availability statement

The data analyzed in this study is subject to the following licenses/restrictions: The authors do not possess full ownership of the database (only partial). Moreover, the original database contains personal data. Requests to access these datasets should be directed to https://dlazdrowia.uck.pl/.

## Ethics statement

The studies involving humans were approved by Independent Bioethics Committee for Scientific Research of Medical University of Gdansk. The studies were conducted in accordance with the local legislation and institutional requirements. Written informed consent for participation in this study was provided by the participants’ legal guardians/next of kin.

## Author contributions

All authors contributed equally to the study design. JS analyzed and interpreted data, and wrote the manuscript. MB processed the data and supervised the process. AS-S supervised the process, corrected and proofread the manuscript. All authors listed have made a substantial, direct, and intellectual contribution to the work and approved it for publication.
