# Supplementary material for: Long-term effects of 12-month integrated weight-loss programme for children with excess body weight- who benefits most?
Source: Front Endocrinol (Lausanne). 2023 Nov 3;14:1221343. doi: 10.3389/fendo.2023.1221343 (PMC10656687; doi:10.3389/fendo.2023.1221343)
Supplement: Supplementary file 1 [file Table_1.docx]

Table S1. *ANCOVA of change in BMI SDS from baseline to follow-up in subgroups by age and sex*

| Subpopulation: Younger | | | | | | | |
| --- | --- | --- | --- | --- | --- | --- | --- |
|  | Baseline | | Follow-up | | Change from baseline | | |
| Group | N | Mean (SD) | N | Mean (SD) | N | Mean (SD) | LS Mean (95% CI)^a^ |
| No participation | 562 | 1.4 ( 0.38) | 562 | 1.4 ( 0.51) | 562 | 0.0 ( 0.41) | 0.01 (-0.03, 0.04) |
| Full participation | 122 | 1.6 ( 0.35) | 122 | 1.5 ( 0.49) | 122 | -0.1 ( 0.40) | -0.10 (-0.17,-0.03) |
| Comparison | | | Difference in LS Mean (95% CI)^a^ | | | | p-value |
| Full participation - No participation | | | -0.11 (-0.19,-0.03) | | | | 0.010 |
| Subpopulation: Older | | | | | | | |
|  | Baseline | | Follow-up | | Change from baseline | | |
| Group | N | Mean (SD) | N | Mean (SD) | N | Mean (SD) | LS Mean (95% CI)^a^ |
| No participation | 329 | 1.4 ( 0.29) | 329 | 1.3 ( 0.64) | 329 | -0.0 ( 0.58) | -0.04 (-0.11, 0.02) |
| Full participation | 119 | 1.5 ( 0.31) | 119 | 1.4 ( 0.59) | 119 | -0.1 ( 0.51) | -0.08 (-0.18, 0.03) |
| Comparison | | | Difference in LS Mean (95% CI)^a^ | | | | p-value |
| Full participation - No participation | | | -0.03 (-0.15, 0.09) | | | | 0.603 |
| Subpopulation: Female | | | | | | | |
|  | Baseline | | Follow-up | | Change from baseline | | |
| Group | N | Mean (SD) | N | Mean (SD) | N | Mean (SD) | LS Mean (95% CI)^a^ |
| No participation | 451 | 1.4 ( 0.37) | 451 | 1.4 ( 0.61) | 451 | 0.0 ( 0.52) | 0.01 (-0.04, 0.05) |
| Full participation | 122 | 1.5 ( 0.33) | 122 | 1.4 ( 0.57) | 122 | -0.1 ( 0.48) | -0.10 (-0.19,-0.00) |
| Comparison | | | Difference in LS Mean (95% CI)^a^ | | | | p-value |
| Full participation - No participation | | | -0.10 (-0.21, 0.00) | | | | 0.054 |
| Subpopulation: Male | | | | | | | |
|  | Baseline | | Follow-up | | Change from baseline | | |
| Group | N | Mean (SD) | N | Mean (SD) | N | Mean (SD) | LS Mean (95% CI)^a^ |
| No participation | 440 | 1.4 ( 0.33) | 440 | 1.4 ( 0.51) | 440 | -0.0 ( 0.44) | -0.03 (-0.07, 0.01) |
| Full participation | 119 | 1.6 ( 0.34) | 119 | 1.5 ( 0.52) | 119 | -0.1 ( 0.44) | -0.07 (-0.15, 0.01) |
| Comparison | | | Difference in LS Mean (95% CI)^a^ | | | | p-value |
| Full participation - No participation | | | -0.04 (-0.13, 0.05) | | | | 0.401 |
| Subpopulation: Younger & Female | | | | | | | |
|  | Baseline | | Follow-up | | Change from baseline | | |
| Group | N | Mean (SD) | N | Mean (SD) | N | Mean (SD) | LS Mean (95% CI)^a^ |
| No participation | 297 | 1.3 ( 0.40) | 297 | 1.4 ( 0.55) | 297 | 0.1 ( 0.43) | 0.05 ( 0.00, 0.10) |
| Full participation | 72 | 1.6 ( 0.35) | 72 | 1.5 ( 0.52) | 72 | -0.1 ( 0.40) | -0.08 (-0.18, 0.02) |
| Comparison | | | Difference in LS Mean (95% CI)^a^ | | | | p-value |
| Full participation - No participation | | | -0.13 (-0.24,-0.02) | | | | 0.025 |
| Subpopulation: Younger & Male | | | | | | | |
|  | Baseline | | Follow-up | | Change from baseline | | |
| Group | N | Mean (SD) | N | Mean (SD) | N | Mean (SD) | LS Mean (95% CI)^a^ |
| No participation | 265 | 1.4 ( 0.36) | 265 | 1.4 ( 0.47) | 265 | -0.0 ( 0.39) | -0.04 (-0.09, 0.01) |
| Full participation | 50 | 1.6 ( 0.34) | 50 | 1.5 ( 0.46) | 50 | -0.2 ( 0.40) | -0.13 (-0.24,-0.03) |
| Comparison | | | Difference in LS Mean (95% CI)^a^ | | | | p-value |
| Full participation - No participation | | | -0.09 (-0.21, 0.03) | | | | 0.124 |
| Subpopulation: Older & Female | | | | | | | |
|  | Baseline | | Follow-up | | Change from baseline | | |
| Group | N | Mean (SD) | N | Mean (SD) | N | Mean (SD) | LS Mean (95% CI)^a^ |
| No participation | 154 | 1.4 ( 0.30) | 154 | 1.3 ( 0.70) | 154 | -0.1 ( 0.64) | -0.08 (-0.18, 0.02) |
| Full participation | 50 | 1.5 ( 0.28) | 50 | 1.3 ( 0.62) | 50 | -0.1 ( 0.57) | -0.12 (-0.30, 0.06) |
| Comparison | | | Difference in LS Mean (95% CI)^a^ | | | | p-value |
| Full participation - No participation | | | -0.04 (-0.25, 0.16) | | | | 0.677 |
| Subpopulation: Older & Male | | | | | | | |
|  | Baseline | | Follow-up | | Change from baseline | | |
| Group | N | Mean (SD) | N | Mean (SD) | N | Mean (SD) | LS Mean (95% CI)^a^ |
| No participation | 175 | 1.4 ( 0.27) | 175 | 1.4 ( 0.58) | 175 | -0.0 ( 0.52) | -0.02 (-0.09, 0.06) |
| Full participation | 69 | 1.5 ( 0.33) | 69 | 1.5 ( 0.56) | 69 | -0.0 ( 0.45) | -0.03 (-0.15, 0.09) |
| Comparison | | | Difference in LS Mean (95% CI)^a^ | | | | p-value |
| Full participation - No participation | | | -0.02 (-0.16, 0.13) | | | | 0.822 |
| ^a^Based on an ANCOVA model after adjusting baseline BMI SDS and observation time. ANCOVA = Analysis of Covariance, CI = Confidence Interval, LS = Least Squares, SD = Standard Deviation | | | | | | | |

Table S2. *ANCOVA of change in SBP SDS from baseline to follow-up in subgroups by age and sex*

| Subpopulation: Younger | | | | | | | |
| --- | --- | --- | --- | --- | --- | --- | --- |
|  | Baseline | | Follow-up | | Change from baseline | | |
| Group | N | Mean (SD) | N | Mean (SD) | N | Mean (SD) | LS Mean (95% CI)^a^ |
| No participation | 562 | 0.3 ( 0.91) | 562 | 0.3 ( 0.99) | 562 | -0.1 ( 1.14) | -0.06 (-0.14, 0.02) |
| Full participation | 122 | 0.3 ( 0.88) | 122 | 0.2 ( 1.01) | 122 | -0.1 ( 1.17) | -0.17 (-0.34, 0.00) |
| Comparison | | | Difference in LS Mean (95% CI)^a^ | | | | p-value |
| Full participation - No participation | | | -0.11 (-0.30, 0.08) | | | | 0.253 |
| Subpopulation: Older | | | | | | | |
|  | Baseline | | Follow-up | | Change from baseline | | |
| Group | N | Mean (SD) | N | Mean (SD) | N | Mean (SD) | LS Mean (95% CI)^a^ |
| No participation | 329 | 0.6 ( 0.97) | 329 | 1.1 ( 1.02) | 329 | 0.5 ( 1.09) | 0.51 ( 0.41, 0.61) |
| Full participation | 119 | 0.4 ( 0.92) | 119 | 1.0 ( 1.05) | 119 | 0.6 ( 1.06) | 0.48 ( 0.31, 0.65) |
| Comparison | | | Difference in LS Mean (95% CI)^a^ | | | | p-value |
| Full participation - No participation | | | -0.03 (-0.23, 0.17) | | | | 0.769 |
| Subpopulation: Female | | | | | | | |
|  | Baseline | | Follow-up | | Change from baseline | | |
| Group | N | Mean (SD) | N | Mean (SD) | N | Mean (SD) | LS Mean (95% CI)^a^ |
| No participation | 451 | 0.4 ( 0.95) | 451 | 0.5 ( 1.01) | 451 | 0.1 ( 1.11) | 0.15 ( 0.06, 0.23) |
| Full participation | 122 | 0.2 ( 0.89) | 122 | 0.4 ( 1.07) | 122 | 0.1 ( 1.15) | 0.05 (-0.12, 0.22) |
| Comparison | | | Difference in LS Mean (95% CI)^a^ | | | | p-value |
| Full participation - No participation | | | -0.10 (-0.29, 0.10) | | | | 0.329 |
| Subpopulation: Male | | | | | | | |
|  | Baseline | | Follow-up | | Change from baseline | | |
| Group | N | Mean (SD) | N | Mean (SD) | N | Mean (SD) | LS Mean (95% CI)^a^ |
| No participation | 440 | 0.5 ( 0.93) | 440 | 0.7 ( 1.13) | 440 | 0.2 ( 1.20) | 0.16 ( 0.06, 0.26) |
| Full participation | 119 | 0.5 ( 0.90) | 119 | 0.8 ( 1.12) | 119 | 0.3 ( 1.18) | 0.26 ( 0.07, 0.45) |
| Comparison | | | Difference in LS Mean (95% CI)^a^ | | | | p-value |
| Full participation - No participation | | | 0.10 (-0.12, 0.31) | | | | 0.377 |
| Subpopulation: Younger & Female | | | | | | | |
|  | Baseline | | Follow-up | | Change from baseline | | |
| Group | N | Mean (SD) | N | Mean (SD) | N | Mean (SD) | LS Mean (95% CI)^a^ |
| No participation | 297 | 0.3 ( 0.90) | 297 | 0.3 ( 0.99) | 297 | 0.0 ( 1.14) | 0.02 (-0.09, 0.13) |
| Full participation | 72 | 0.2 ( 0.86) | 72 | -0.0 ( 1.02) | 72 | -0.2 ( 1.13) | -0.26 (-0.48,-0.03) |
| Comparison | | | Difference in LS Mean (95% CI)^a^ | | | | p-value |
| Full participation - No participation | | | -0.27 (-0.52,-0.03) | | | | 0.031 |
| Subpopulation: Younger & Male | | | | | | | |
|  | Baseline | | Follow-up | | Change from baseline | | |
| Group | N | Mean (SD) | N | Mean (SD) | N | Mean (SD) | LS Mean (95% CI)^a^ |
| No participation | 265 | 0.4 ( 0.92) | 265 | 0.3 ( 0.99) | 265 | -0.2 ( 1.15) | -0.15 (-0.26,-0.03) |
| Full participation | 50 | 0.4 ( 0.90) | 50 | 0.4 ( 0.95) | 50 | 0.0 ( 1.24) | -0.03 (-0.29, 0.24) |
| Comparison | | | Difference in LS Mean (95% CI)^a^ | | | | p-value |
| Full participation - No participation | | | 0.12 (-0.17, 0.41) | | | | 0.420 |
| Subpopulation: Older & Female | | | | | | | |
|  | Baseline | | Follow-up | | Change from baseline | | |
| Group | N | Mean (SD) | N | Mean (SD) | N | Mean (SD) | LS Mean (95% CI)^a^ |
| No participation | 154 | 0.6 ( 1.00) | 154 | 0.9 ( 0.92) | 154 | 0.3 ( 1.03) | 0.39 ( 0.26, 0.52) |
| Full participation | 50 | 0.3 ( 0.95) | 50 | 0.9 ( 0.88) | 50 | 0.7 ( 1.00) | 0.50 ( 0.26, 0.73) |
| Comparison | | | Difference in LS Mean (95% CI)^a^ | | | | p-value |
| Full participation - No participation | | | 0.11 (-0.16, 0.38) | | | | 0.425 |
| Subpopulation: Older & Male | | | | | | | |
|  | Baseline | | Follow-up | | Change from baseline | | |
| Group | N | Mean (SD) | N | Mean (SD) | N | Mean (SD) | LS Mean (95% CI)^a^ |
| No participation | 175 | 0.6 ( 0.94) | 175 | 1.2 ( 1.09) | 175 | 0.6 ( 1.13) | 0.62 ( 0.47, 0.78) |
| Full participation | 69 | 0.5 ( 0.90) | 69 | 1.0 ( 1.17) | 69 | 0.5 ( 1.10) | 0.46 ( 0.22, 0.70) |
| Comparison | | | Difference in LS Mean (95% CI)^a^ | | | | p-value |
| Full participation - No participation | | | -0.17 (-0.45, 0.12) | | | | 0.254 |
| ^a^Based on an ANCOVA model after adjusting baseline SBP SDS and observation time. ANCOVA = Analysis of Covariance, CI = Confidence Interval, LS = Least Squares, SD = Standard Deviation | | | | | | | |

Table S3. *ANCOVA of change in DBP SDS from baseline to follow-up in subgroups by age and sex*

| Subpopulation: Younger | | | | | | | |
| --- | --- | --- | --- | --- | --- | --- | --- |
|  | Baseline | | Follow-up | | Change from baseline | | |
| Group | N | Mean (SD) | N | Mean (SD) | N | Mean (SD) | LS Mean (95% CI)^a^ |
| No participation | 562 | 1.2 ( 0.86) | 562 | 1.1 ( 0.97) | 562 | -0.0 ( 1.16) | -0.02 (-0.10, 0.06) |
| Full participation | 122 | 1.1 ( 0.86) | 122 | 1.0 ( 0.99) | 122 | -0.1 ( 1.20) | -0.12 (-0.29, 0.05) |
| Comparison | | | Difference in LS Mean (95% CI)^a^ | | | | p-value |
| Full participation - No participation | | | -0.10 (-0.29, 0.09) | | | | 0.293 |
| Subpopulation: Older | | | | | | | |
|  | Baseline | | Follow-up | | Change from baseline | | |
| Group | N | Mean (SD) | N | Mean (SD) | N | Mean (SD) | LS Mean (95% CI)^a^ |
| No participation | 329 | 1.2 ( 0.95) | 329 | 0.6 ( 1.14) | 329 | -0.6 ( 1.23) | -0.57 (-0.69,-0.46) |
| Full participation | 119 | 1.1 ( 0.98) | 119 | 0.4 ( 1.09) | 119 | -0.7 ( 1.24) | -0.79 (-0.99,-0.60) |
| Comparison | | | Difference in LS Mean (95% CI)^a^ | | | | p-value |
| Full participation - No participation | | | -0.22 (-0.45, 0.00) | | | | 0.053 |
| Subpopulation: Female | | | | | | | |
|  | Baseline | | Follow-up | | Change from baseline | | |
| Group | N | Mean (SD) | N | Mean (SD) | N | Mean (SD) | LS Mean (95% CI)^a^ |
| No participation | 451 | 1.1 ( 0.91) | 451 | 0.9 ( 1.05) | 451 | -0.2 ( 1.23) | -0.20 (-0.29,-0.11) |
| Full participation | 122 | 1.1 ( 0.93) | 122 | 0.7 ( 0.96) | 122 | -0.4 ( 1.16) | -0.41 (-0.59,-0.24) |
| Comparison | | | Difference in LS Mean (95% CI)^a^ | | | | p-value |
| Full participation - No participation | | | -0.21 (-0.41,-0.01) | | | | 0.038 |
| Subpopulation: Male | | | | | | | |
|  | Baseline | | Follow-up | | Change from baseline | | |
| Group | N | Mean (SD) | N | Mean (SD) | N | Mean (SD) | LS Mean (95% CI)^a^ |
| No participation | 440 | 1.2 ( 0.87) | 440 | 0.9 ( 1.09) | 440 | -0.3 ( 1.20) | -0.25 (-0.35,-0.15) |
| Full participation | 119 | 1.1 ( 0.92) | 119 | 0.7 ( 1.22) | 119 | -0.4 ( 1.36) | -0.48 (-0.68,-0.28) |
| Comparison | | | Difference in LS Mean (95% CI)^a^ | | | | p-value |
| Full participation - No participation | | | -0.23 (-0.45,-0.01) | | | | 0.041 |
| Subpopulation: Younger & Female | | | | | | | |
|  | Baseline | | Follow-up | | Change from baseline | | |
| Group | N | Mean (SD) | N | Mean (SD) | N | Mean (SD) | LS Mean (95% CI)^a^ |
| No participation | 297 | 1.1 ( 0.86) | 297 | 1.1 ( 0.96) | 297 | -0.0 ( 1.18) | 0.00 (-0.10, 0.11) |
| Full participation | 72 | 1.1 ( 0.82) | 72 | 0.9 ( 0.94) | 72 | -0.2 ( 1.09) | -0.25 (-0.46,-0.03) |
| Comparison | | | Difference in LS Mean (95% CI)^a^ | | | | p-value |
| Full participation - No participation | | | -0.25 (-0.49,-0.01) | | | | 0.043 |
| Subpopulation: Younger & Male | | | | | | | |
|  | Baseline | | Follow-up | | Change from baseline | | |
| Group | N | Mean (SD) | N | Mean (SD) | N | Mean (SD) | LS Mean (95% CI)^a^ |
| No participation | 265 | 1.2 ( 0.84) | 265 | 1.2 ( 0.98) | 265 | -0.1 ( 1.13) | -0.05 (-0.16, 0.07) |
| Full participation | 50 | 1.1 ( 0.93) | 50 | 1.3 ( 1.03) | 50 | 0.2 ( 1.33) | 0.08 (-0.19, 0.35) |
| Comparison | | | Difference in LS Mean (95% CI)^a^ | | | | p-value |
| Full participation - No participation | | | 0.12 (-0.17, 0.41) | | | | 0.421 |
| Subpopulation: Older & Female | | | | | | | |
|  | Baseline | | Follow-up | | Change from baseline | | |
| Group | N | Mean (SD) | N | Mean (SD) | N | Mean (SD) | LS Mean (95% CI)^a^ |
| No participation | 154 | 1.2 ( 1.01) | 154 | 0.6 ( 1.14) | 154 | -0.6 ( 1.22) | -0.59 (-0.75,-0.42) |
| Full participation | 50 | 1.1 ( 1.07) | 50 | 0.5 ( 0.95) | 50 | -0.6 ( 1.25) | -0.67 (-0.96,-0.38) |
| Comparison | | | Difference in LS Mean (95% CI)^a^ | | | | p-value |
| Full participation - No participation | | | -0.08 (-0.42, 0.26) | | | | 0.636 |
| Subpopulation: Older & Male | | | | | | | |
|  | Baseline | | Follow-up | | Change from baseline | | |
| Group | N | Mean (SD) | N | Mean (SD) | N | Mean (SD) | LS Mean (95% CI)^a^ |
| No participation | 175 | 1.1 ( 0.91) | 175 | 0.6 ( 1.15) | 175 | -0.6 ( 1.24) | -0.56 (-0.72,-0.40) |
| Full participation | 69 | 1.1 ( 0.91) | 69 | 0.3 ( 1.17) | 69 | -0.8 ( 1.23) | -0.88 (-1.14,-0.62) |
| Comparison | | | Difference in LS Mean (95% CI)^a^ | | | | p-value |
| Full participation - No participation | | | -0.32 (-0.62,-0.01) | | | | 0.042 |
| ^a^Based on an ANCOVA model after adjusting baseline DBP SDS and observation time. ANCOVA = Analysis of Covariance, CI = Confidence Interval, LS = Least Squares, SD = Standard Deviation | | | | | | | |

Table S4. *ANCOVA of change in post-exercise HR (KPRT) from baseline to follow-up in subgroups by age and sex*

| Subpopulation: Younger | | | | | | | |
| --- | --- | --- | --- | --- | --- | --- | --- |
|  | Baseline | | Follow-up | | Change from baseline | | |
| Group | N | Mean (SD) | N | Mean (SD) | N | Mean (SD) | LS Mean (95% CI)^a^ |
| No participation | 344 | 119.1 (12.91) | 344 | 119.0 (16.20) | 344 | -0.1 (15.62) | -0.47 (-2.01, 1.06) |
| Full participation | 77 | 122.5 (14.05) | 77 | 121.5 (14.70) | 77 | -1.0 (17.05) | 0.81 (-2.48, 4.10) |
| Comparison | | | Difference in LS Mean (95% CI)^a^ | | | | p-value |
| Full participation - No participation | | | 1.28 (-2.36, 4.92) | | | | 0.490 |
| Subpopulation: Older | | | | | | | |
|  | Baseline | | Follow-up | | Change from baseline | | |
| Group | N | Mean (SD) | N | Mean (SD) | N | Mean (SD) | LS Mean (95% CI)^a^ |
| No participation | 192 | 126.2 (16.82) | 192 | 120.1 (21.01) | 192 | -6.2 (24.08) | -5.61 (-8.65,-2.57) |
| Full participation | 77 | 124.7 (24.97) | 77 | 120.9 (22.39) | 77 | -3.8 (34.43) | -5.19 (-10.01,-0.37) |
| Comparison | | | Difference in LS Mean (95% CI)^a^ | | | | p-value |
| Full participation - No participation | | | 0.42 (-5.28, 6.12) | | | | 0.885 |
| Subpopulation: Female | | | | | | | |
|  | Baseline | | Follow-up | | Change from baseline | | |
| Group | N | Mean (SD) | N | Mean (SD) | N | Mean (SD) | LS Mean (95% CI)^a^ |
| No participation | 260 | 122.8 (16.15) | 260 | 122.4 (17.82) | 260 | -0.4 (18.75) | -1.30 (-3.37, 0.76) |
| Full participation | 74 | 128.9 (14.93) | 74 | 124.9 (17.75) | 74 | -4.0 (21.96) | -0.92 (-4.83, 2.99) |
| Comparison | | | Difference in LS Mean (95% CI)^a^ | | | | p-value |
| Full participation - No participation | | | 0.38 (-4.06, 4.82) | | | | 867 |
| Subpopulation: Male | | | | | | | |
|  | Baseline | | Follow-up | | Change from baseline | | |
| Group | N | Mean (SD) | N | Mean (SD) | N | Mean (SD) | LS Mean (95% CI)^a^ |
| No participation | 276 | 120.5 (13.38) | 276 | 116.6 (17.85) | 276 | -4.0 (19.67) | -3.59 (-5.75,-1.44) |
| Full participation | 80 | 118.8 (23.17) | 80 | 117.8 (19.35) | 80 | -1.0 (31.21) | -2.24 (-6.29, 1.80) |
| Comparison | | | Difference in LS Mean (95% CI)^a^ | | | | p-value |
| Full participation - No participation | | | 1.35 (-3.25, 5.95) | | | | 0.565 |
| aBased on an ANCOVA model after adjusting baseline KPRT and observation time.  ANCOVA = Analysis of Covariance, CI = Confidence Interval, LS = Least Squares, SD = Standard Deviation | | | | | | | |
